# Comparative Efficacy of First-Line Therapeutic Options for ES-SCLC: An Indirect Comparison Using IPDfromKM-Reconstructed Individual Patient Data

**DOI:** 10.3390/cancers18121869

**Published:** 2026-06-08

**Authors:** Lorenzo Gasperoni, Tiziano Lupi, Luna Del Bono, Valentina Polo, Andrea Messori, Vera Damuzzo

**Affiliations:** 1 Pharmaceutical Department, USL Toscana Centro, 59100 Prato, Italy; lorenzo.gasperoni@uslcentro.toscana.it; 2 Hospital Pharmacy Unit, “SS. Annunziata” Hospital, ASL 02, 66100 Chieti, Italy; 3 Italian Society of Clinical Pharmacy and Therapeutics (SIFaCT), 10123 Turin, Italy; vera.damuzzo@aulss2.veneto.it; 4 Department of Pharmacy, School of Specialization in Hospital Pharmacy, University of Pisa, 56126 Pisa, Italy; l.delbono3@studenti.unipi.it; 5 Oncology Unit, AULSS 2 Marca Trevigiana, 33100 Treviso, Italy; valentina.polo@aulss2.veneto.it; 6 Osservatorio Innovazione Study Group, 50134 Firenze, Italy; 7 Hospital Pharmacy Department, AULSS 2 Marca Trevigiana, 31100 Treviso, Italy

**Keywords:** extensive-small cell lung cancer, indirect comparison, restricted mean survival time

## Abstract

Background: Extensive-stage small cell lung cancer (ES-SCLC) carries a poor prognosis, with fewer than 7% of patients surviving for five years. While immune checkpoint inhibitors (ICIs) combined with platinum–etoposide have reshaped first-line treatment, no head-to-head trials exist comparing available regimens, leaving the optimal therapeutic choice undefined. Methods: A systematic literature search identified phase III randomized controlled trials (RCTs) evaluating first-line ICI-based regimens in ES-SCLC. Individual patient data (IPD) were reconstructed from Kaplan–Meier curves using the IPDfromKM algorithm. Indirect treatment comparisons were performed using Cox proportional hazards models, with chemotherapy alone as the common comparator. The indirect comparison of tiragolumab efficacy was unanchored due to the design of the SKYSCRAPER-02 study. Restricted mean survival time (RMST), truncated at 27 months, was calculated as an additional measure of treatment effect. Results: Six RCTs were included. All ICI-based regimens improved overall survival (OS) compared to chemotherapy alone, except ipilimumab (HR 0.97, 95% CI 0.86–1.09). Serplulimab demonstrated the most favorable OS benefit (HR 0.55, 95% CI 0.48–0.64; RMST 16.73 months), representing a gain of approximately 4 months over chemotherapy alone and 1.8–2.3 months over atezolizumab and durvalumab. The addition of tiragolumab to atezolizumab plus chemotherapy yielded no significant advantage over atezolizumab alone. Conclusions: This IPD-based indirect comparison suggests that serplulimab plus chemotherapy may offer the most favorable OS estimates S benefit among first-line ICI regimens for ES-SCLC, while durvalumab and atezolizumab remain effective standards of care. These findings are hypothesis-generating and highlight the need for prospective head-to-head comparative studies.

## Simple Summary

This research tackles a major gap in treating extensive-stage small cell lung cancer (ES-SCLC), a highly aggressive disease in which fewer than 7% of patients survive for five years. There are currently no direct clinical trials comparing the efficacy of new immunotherapy drugs, such as serplulimab or atezolizumab, when added to standard chemotherapy. This leaves oncologists uncertain about the most effective treatment. We aim to indirectly compare these options by analyzing data from six major trials, showing that serplulimab plus chemotherapy extends life by approximately four months more than chemotherapy alone (and two months more than competitor drugs). These findings could encourage the research community to conduct head-to-head trials, prioritize serplulimab in guidelines, and investigate why it is more effective, ultimately leading to improved first-line treatment options for patients.

## 1. Introduction

Small cell lung cancer (SCLC) represents roughly 13–17% of lung cancer cases and is marked by rapid proliferation, a high growth fraction, and early dissemination with widespread metastases [1,2]. SCLC is typically divided into two clinical categories: limited-stage SCLC (LS-SCLC) and extensive-stage SCLC (ES-SCLC). LS-SCLC refers to tumors confined to one hemithorax (frequently involving regional lymph nodes), and it is usually treatable within a single radiotherapy field. Conversely, ES-SCLC involves spread beyond the hemithorax, with distant metastatic involvement of multiple anatomical sites [3]. ES-SCLC comprises approximately 70% of SCLC cases. Additionally, SCLC is often linked to paraneoplastic syndromes, predominantly neurologic and endocrine manifestations [4]. Despite treatment advances, the prognosis remains poor, with fewer than 7% of patients alive five years after diagnosis [2,3,4,5,6,7].

Although initial response rates are high, resistance frequently occurs, and the median overall survival (OS) with chemotherapy alone has historically remained approximately 10–12 months [8]. In line with non-small cell lung cancer (NSCLC), the introduction of immune checkpoint inhibitors (ICIs) to the treatment algorithm for this cancer expanded available pharmacological options and created a new field of SCLC treatment [9,10]. The advent of ICIs targeting programmed cell death 1 (PD-1), programmed cell death ligand 1 (PD-L1), and cytotoxic T lymphocyte-associated protein 4 (CTLA-4)—including atezolizumab, pembrolizumab, ipilimumab, durvalumab, and serplulimab—has demonstrated improvements in overall survival (OS) and progression-free survival (PFS) in patients with SCLC, alongside a generally more favorable toxicity profile relative to conventional chemotherapy [11,12,13,14,15]. Incorporating PD-1/PD-L1 blockade into platinum–etoposide regimens has transformed the first-line therapeutic paradigm for ES-SCLC; nevertheless, OS benefits across immune checkpoint strategies vary considerably and do not follow a strictly linear pattern.

In the IMpower133 and CASPIAN trials, the addition of atezolizumab or durvalumab to platinum–etoposide therapy demonstrated to improve OS compared to chemotherapy alone (median OS of 12.3 vs. 10.3 months and HR 0.76, 95% CI 0.60–0.95 and median OS of 12.9 vs. 10.5 months and HR 0.75, 95% CI 0.62–0.91, respectively) [16,17]. In contrast, the KEYNOTE-604 trial showed that pembrolizumab plus platinum–etoposide yielded an OS hazard ratio favoring immunotherapy that failed to reach the prespecified significance boundary (median OS 10.8 vs. 9.7 months; HR 0.80, 95% CI 0.64–0.98; *p* = 0.06) [13]. Likewise, targeting CTLA-4 with ipilimumab did not extend OS in the CA184-156 trial (median OS 11.0 vs. 10.9 months; HR 0.94, 95% CI 0.81–1.09; *p* = 0.3775) [14]. More recently, novel combinations of immunomodulatory agents have been added to a chemotherapy backbone in an effort to improve OS, such as the anti-TIGIT monoclonal antibody tiragolumab combined with the anti-PDL-1 agent atezolizumab. Yet, as shown in the SKYSCRAPER-02 study, this dual immunotherapeutic approach did not succeed (median OS 13.11 vs. 13.14 months; HR 1.14, 95% CI 0.90–1.44) [18]. An alternative strategy to enhance ICI efficacy involves designing new anti-PD-1 monoclonal antibodies. Serplulimab, for instance, has demonstrated superior affinity for the PD-1 receptor and a more potent immunomodulatory effect within the tumor microenvironment [19].

In the ASTRUM-005 study, serplulimab plus chemotherapy significantly prolonged OS with an apparently greater OS benefit compared to other ICIs (median OS 15.4 vs. 10.9 months; HR 0.63, 95% CI 0.49–0.82) [12,20]. Overall, the definitive impact of immune checkpoint blockade in ES-SCLC remains uncertain due to the variable efficacy and safety of the different approaches. Furthermore, the relative efficacy of various therapeutic options has not been evaluated in direct head-to-head trials. Therefore, the most appropriate choice remains undefined. In this rapidly evolving landscape, a comparative assessment of these regimens is important to inform clinical and regulatory decision-making. In this study, a statistical method and computational tool, named IPDfromKM, has been used to reconstruct individual patient data (IPD) from Kaplan–Meier (KM) curves, enabling cross-trial comparisons between first-line therapeutic options for ES-SCLC, as direct comparisons in randomized controlled trials (RCTs) are unavailable [21,22,23].

## 2. Materials and Methods

### 2.1. Literature Search

We conducted a systematic search of the PubMed database to identify RCTs that were relevant to our analysis. The last search was performed on 26 January 2026. The search strategy was as follows: [(“ES-SCLC” OR “es-SCLC” OR “Extensive-Stage Small Cell Lung Cancer” OR “Extensive-Stage Small-Cell Lung Cancer” OR “Extensive-Stage SCLC”) AND (“first-line” OR “first line” OR “untreated”)]. The selection process adhered to the PRISMA guidelines [24]. The inclusion criteria were as follows: (a) phase III RCT; (b) first-line treatment of ES-SCLC; (c) ICI treatment; (d) availability of OS outcomes; and (e) data presented through KM survival curves. Studies that only enrolled Asian patients were excluded. To avoid including the same patients from the same trial more than once, the most recent publication from each trial was selected. The entire procedure of literature search and trial selection was independently performed in duplicate by two investigators. The literature search was conducted based on the 2020 version of the PRISMA algorithm [24]. The PRISMA checklist was compiled and is available from the Supplementary Materials S1. The protocol for this systematic review was registered in the PROSPERO database (https://www.crd.york.ac.uk/PROSPERO/view/CRD420261375409, accessed 4 June 2026).

### 2.2. Reconstruction of Individual Patient Data

IPD were reconstructed from the KM survival curves of the treatment and control arms reported in the selected randomized controlled trials (RCTs) using the IPDfromKM method [21]. KM curves were digitized with WebPlotDigitizer (version 4.7; https://apps.automeris.io/wpd/; accessed 1 February 2026; distance = 20, Δx = 15, Δy = 15). The extracted X–Y coordinates, together with the reported numbers of enrolled patients and events, were subsequently processed using the IPDfromKM package (version 1.2.3.0; last updated 22 March 2022; https://www.trialdesign.org/one-page-shell.html#IPDfromKM; accessed 2 February 2026). This approach generated reconstructed datasets containing survival times, defined as the interval from trial enrolment to the last available follow-up, and patient outcomes, categorized as alive/censored or deceased. Reconstructed IPD were obtained for each RCT arm. The entire procedure, including curve digitization and IPD reconstruction, was independently performed in duplicate by two investigators, and the resulting datasets were compared for consistency to minimize the risk of manual errors.

### 2.3. Study Design and Data Analysis

This analysis aimed to evaluate the relative efficacy of first-line ICI treatments for patients with ES-SCLC, with OS as the primary endpoint. We focused our analysis on overall survival because OS represents the most robust and clinically meaningful endpoint in ES-SCLC, being less susceptible than PFS to assessment timing and investigator-related variability. The regimens evaluated included serplulimab, durvalumab, ipilimumab, pembrolizumab, atezolizumab combined with chemotherapy, and a treatment combining tiragolumab and atezolizumab with chemotherapy.

KM curves for OS were reconstructed for each trial arm. For comparative analyses, a placebo was adopted as the common comparator across the included trials. All patients receiving placebo + chemotherapy across the included studies were pooled and analyzed together to constitute the control group. As the control group in the SKYSCRAPER-02 study consisted of an active treatment (atezolizumab plus chemotherapy), we pooled and analyzed the SKYSCRAPER-02 control arm and the IMpower133 experimental arm together. Even though comparison with other treatment options will be unanchored due to the lack of a common comparator, the experimental arm of the SKYSCRAPER-02 study was included in the analysis. This is because the study explored a triplet regimen of tiragolumab plus atezolizumab plus chemotherapy, which was therefore compared to a doublet of atezolizumab plus chemotherapy.

Treatment effects were estimated using a Cox proportional hazards model, and the results were expressed as hazard ratios (HRs) with 95% confidence intervals (CIs). Assessment of proportional hazards was performed using the Schoenfeld residuals test. The homogeneity of the control groups across the trials was exploratively evaluated using likelihood ratio tests and concordance statistics. Similarly, the same homogeneity test was used for the SKYSCRAPER-02 population control arm and the IMpower133 experimental arm, and the results were pooled. Both arms consisted of atezolizumab plus chemotherapy. Indirect pairwise comparisons among active regimens were also performed using Cox regression models.

Restricted mean survival time (RMST) was calculated as an additional measure of treatment effect for OS, with KM curves truncated at 27 months, which corresponds to the minimum follow-up time available across the included RCTs. All statistical analyses were performed with R software (version 4.5.3).

## 3. Results

**Figure 1:**
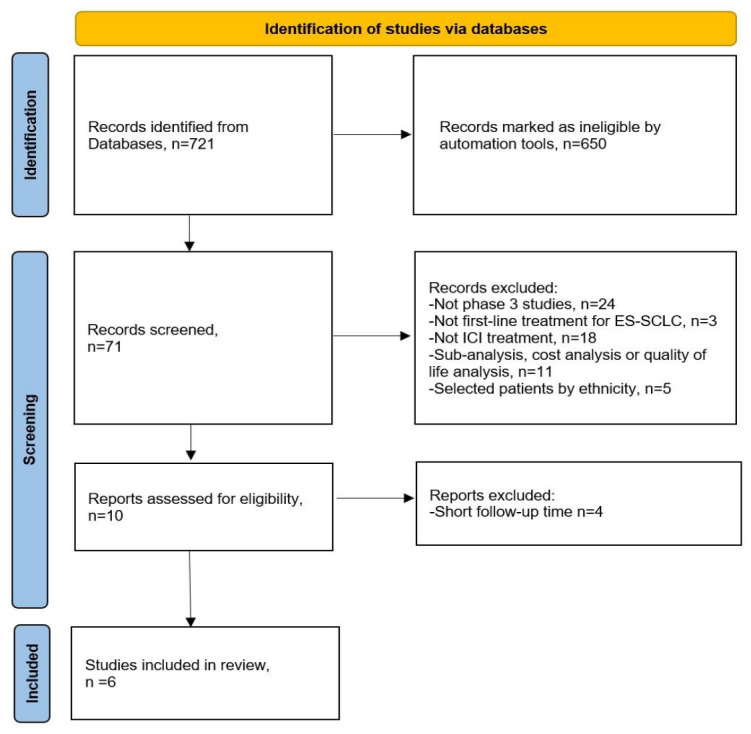
PRISMA 2020 flowchart of the process of trial selection. Abbreviations: ES-SCLC, extensive-stage small cell lung cancer; ICI, immune checkpoint inhibitor.

A comprehensive literature search yielded 721 records, which were screened according to predefined inclusion and exclusion criteria to identify the most recent RCTs evaluating first-line therapy with ICIs plus chemotherapy in patients with ES-SCLC. The selection process, illustrated in Figure 1 in accordance with the PRISMA recommendations, resulted in six RCTs being identified as eligible for an indirect comparison analysis of OS outcomes.

Several major trials have examined immunotherapy combinations with platinum–etoposide chemotherapy in this setting. CASPIAN evaluated durvalumab, KEYNOTE-604 assessed pembrolizumab, and ASTRUM-005 investigated serplulimab, each combined with platinum–etoposide. Additionally, the phase III CA184-156 trial by Reck et al. examined ipilimumab alongside the same chemotherapy backbone. All included studies, except SKYSCRAPER-02, contained a platinum–etoposide chemotherapy control arm, which was used as the common comparator for indirect comparison. Indeed, the SKYSCRAPER-02 RCT explored a triplet regimen of tiragolumab plus atezolizumab plus chemotherapy, which was therefore compared to a doublet of atezolizumab plus chemotherapy. IPD from two trials, IMpower133 and SKYSCRAPER-02, were pooled as they included patients treated with atezolizumab and chemotherapy. Table 1 summarizes the main clinical and demographic characteristics of patients enrolled in the included trials.

**Figure A1:**
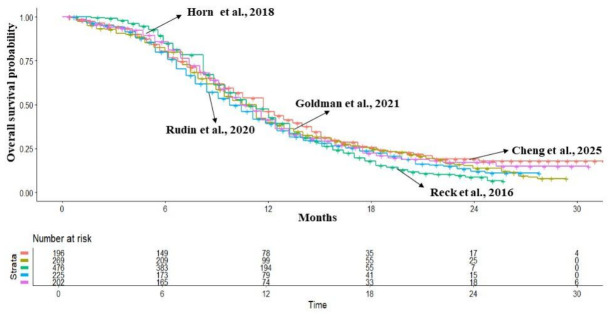
Kaplan–Meier curves generated after reconstructing patient-level data from the control arms of the included trials (platinum-based chemotherapy): Cheng et al. ASTRUM-005 RCT (n = 196; in red [20]); Goldman et al. CASPIAN RCT (n = 269; in gold [17]); Reck et al. CA-184-156 RCT (n = 476; in green [14]); Rudin et al. Keynote-604 RCT (n = 225; in blue [13]); Horn et al. Impower133 RCT (n = 202; in pink [11]); Endpoint: Overall survival, time in months. n, number of patients.

The populations enrolled in the included trials were not entirely homogeneous with regard to ethnicity, smoking status, platinum backbone selection, and the prevalence of baseline brain metastases. The proportion of Asian patients varied markedly across studies, ranging from 13% in CASPIAN to 67% in ASTRUM-005, reflecting differences in geographic recruitment. Similarly, the percentage of current smokers showed considerable variability, spanning from 26% in the ASTRUM-005 study to 92% in the CA184-156 RCT. The use of cisplatin as an alternative to carboplatin also differed between trials (0–34%), and finally, the incidence of baseline brain metastases ranged from 8.5% in IMpower133 to 19% in SKYSCRAPER-02, representing a potential source of prognostic heterogeneity across study populations. In view of the differences in baseline clinical characteristics across trials, we performed a formal heterogeneity analysis to assess whether patients receiving the same therapeutic backbone, i.e., platinum–etoposide chemotherapy, demonstrated comparable OS outcomes across studies. Despite the observed variability in demographic and clinical features, no statistically significant heterogeneity was detected for OS among the control arms (likelihood ratio test = 3.88; 4 degrees of freedom; *p* = 0.4), supporting the assumption of cross-trial comparability. KM curves for the OS of the control arms are reported in Figure A1.

**Figure A2:**
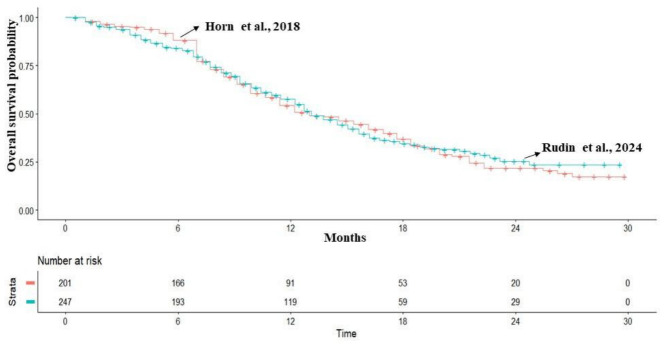
Kaplan–Meier curves generated after reconstructing patient-level data from the control arms of the Skyscraper-002 RCT (Rudin et al. [18], n = 247, in turquoise) and the active arm of the Impower133 RCT (Horn et al. [11], n = 201, in red). Both arms included patients receiving atezolizumab + chemotherapy.

As patients in the control arm of the Skyscraper-02 study received the same treatment as those in the active arm of the Impower133 study (i.e., atezolizumab plus chemotherapy), we performed a heterogeneity test to determine whether the results of the patients from these two studies could be combined. This test demonstrated substantial homogeneity between the two groups (Likelihood ratio test = 0.15 on 1 df, *p* = 0.7), meaning they could be combined for subsequent analyses as a single group of patients treated with atezolizumab plus chemotherapy. Figure A2 shows the KM curves for the OS of the groups of patients treated with atezolizumab + chemotherapy. To evaluate the accuracy of the IPDfromKM method’s reconstruction of patient-level data, we compared the original hazard ratios (HRs) reported in the published trials with those generated by six trial-specific IPDfromKM analyses. To ensure comparability, control arms were not pooled. The results of these six comparisons (see Table A1) showed excellent agreement between the original HR values and those recalculated from the reconstructed patients.

**Figure 2:**
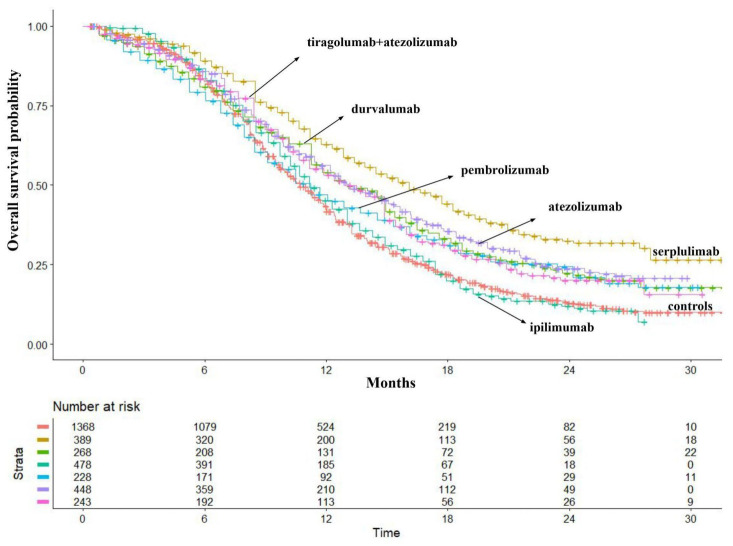
OS comparison of first-line treatments for ES-SCLC. After reconstructing individual patient data (IPD) from six trials, the following OS KM curves were generated for active arms of the RCTs, which combine platinum-based chemotherapy with serplulimab (n = 389; in gold); durvalumab (n = 268; in light green); ipilimumab (n = 478; in dark green); pembrolizumab (n = 228; in light blue); atezolizumab (n = 448, in violet); and tiragolumab + atezolizumab (n = 243; in pink). The control arm (n = 1368; in red) was generated by pooling IPD from the chemotherapy-based control arms. Abbreviations: n, number of patients.

After confirming cross-trial comparability, we ensured proportional hazards using the Schoenfeld residuals test, which showed no evidence of violation globally in the OS analysis of the population (*p* = 0.23). Having confirmed that the methodological prerequisites for the proposed analysis were satisfied, we performed indirect comparisons among the therapeutic options evaluated across the included RCTs. Adding ICIs to chemotherapy demonstrated a statistically significant OS benefit compared to chemotherapy alone across all regimens evaluated. Among the ICIs examined, serplulimab yielded the most favorable OS outcomes when combined with chemotherapy (HR = 0.55, 95% CI: 0.48–0.64). The results are shown in Figure 2 and Supplementary Materials S2.

Inter-treatment comparisons, the results of which are displayed in Table 2, further confirmed the most favorable observed profile of serplulimab plus chemotherapy over all other regimens, including atezolizumab-based (HR = 0.78, 95% CI: 0.64–0.95) and durvalumab-based combinations (HR = 0.74, 95% CI: 0.60–0.91).

These findings were corroborated by restricted mean survival time (RMST) analysis, in which serplulimab plus chemotherapy achieved an RMST of 16.73 months (95% CI: 15.82–17.64), that is approximately 4 months longer than RMST of chemotherapy (RMST = 12.59 months, 95% CI: 12.18–13.00), 2.3 months longer than RMST of atezolizumab plus chemotherapy (RMST = 14.91 months, 95% CI: 14.08–15.74), and 1.8 months longer than RMST of durvalumab plus chemotherapy (RMST = 14.44 months, 95% CI: 13.40–15.49). Results from the RMST analysis are shown in Table 3.

Both durvalumab and atezolizumab, when added to chemotherapy, conferred a modest but significant improvement in OS compared to chemotherapy alone (HR = 0.75, 95% CI: 0.64–0.87 and HR = 0.71, 95% CI: 0.62–0.80, respectively). Inter-treatment comparisons revealed that both agents produced an OS benefit over ipilimumab plus chemotherapy, which demonstrated the least benefit among the regimens evaluated, failing to achieve a statistically significant OS improvement over chemotherapy alone (HR = 0.97, 95% CI: 0.86–1.09). Adding tiragolumab to the atezolizumab plus chemotherapy treatment resulted in an apparent advantage of OS compared to chemotherapy alone (HR = 0.78, 95% CI: 0.66–0.91); however, this combination did not demonstrate any significant advantage over atezolizumab plus chemotherapy (HR = 1.01, 95% CI: 0.90–1.35), suggesting no added benefit from the dual checkpoint blockade strategy. Pembrolizumab plus chemotherapy yielded limited OS improvement relative to other ICI-based regimens, with only a marginal and non-significant difference compared to ipilimumab plus chemotherapy (inter-treatment HR = 0.87, 95% CI: 0.70–1.07).

## 4. Discussion

This indirect comparative analysis, which is based on reconstructed IPD, evaluates the relative efficacy of first-line ICI-based strategies in ES-SCLC, using chemotherapy as the comparator and applying the IPDfromKM method. Our findings suggest that adding ICIs to platinum–etoposide improves OS compared with chemotherapy alone, with meaningful differences emerging between the different agents. Notably, serplulimab plus chemotherapy was associated with the most favorable survival outcomes across both HR and RMST analyses. Our results are broadly consistent with the pivotal randomized trials that established chemo-immunotherapy as the standard of care in ES-SCLC, including IMpower133 and CASPIAN, in which atezolizumab and durvalumab, respectively, demonstrated a modest but statistically significant OS benefit over chemotherapy alone [11,15,16,17]. However, cross-trial comparisons remain challenging due to differences in study populations, follow-up duration, and statistical assumptions. By reconstructing IPD from KM curves, we aimed to address the absence of head-to-head trials and provide a unified comparative framework.

Among the regimens evaluated, serplulimab demonstrated the greatest magnitude of OS benefit. This finding is consistent with the results of the ASTRUM-005 trial, which reported a median OS of 15.4 months, which is longer than that observed in other ICI trials in this setting. The apparent advantage observed in our indirect analysis may reflect several factors. Firstly, serplulimab has been reported to exhibit a high binding affinity for PD-1, as well as enhanced immune modulation within the tumor microenvironment. Secondly, differences in patient selection, subsequent therapies or geographic distribution may have contributed to the improved outcomes. It is worth noting that ASTRUM-005 included a significant proportion of Asian patients and a lower proportion of smokers (26%) compared to other studies, suggesting that ethnic or regional variations in tumor biology, treatment approaches, or supportive care could have impacted survival rates. Nonetheless, in ASTRUM-005, the survival benefit associated with serplulimab plus chemotherapy was consistent across the prespecified subgroups, including smoking history and ethnicity, supporting the robustness of the treatment effect across clinically relevant patient populations. Moreover, although immunotherapy is generally considered less effective in never-smokers in other thoracic malignancies such as NSCLC, the authors of ASTRUM-005 suggested that a proportion of never-smoker SCLC patients may nevertheless have been exposed to passive smoking, potentially contributing to tumour immunogenicity. Hence, although we excluded trials enrolling exclusively Asian populations and formally tested control arm homogeneity, residual confounding factors cannot be entirely ruled out. On the other hand, durvalumab and atezolizumab demonstrated similar efficacy, both providing a clinically significant, albeit quantitatively modest, survival advantage over chemotherapy alone. These results reinforce their established role as reference standards in first-line ES-SCLC treatment. In contrast, ipilimumab failed to demonstrate a survival benefit, which is consistent with the negative findings of the CA184-156 trial. This suggests that CTLA-4 inhibition does not provide an additional benefit in this disease context when combined with platinum–etoposide. The dual-checkpoint blockade strategy combining tiragolumab with atezolizumab did not demonstrate an additional survival benefit over atezolizumab plus chemotherapy. This finding is consistent with the negative results of SKYSCRAPER-02 and indicates that intensifying immune modulation beyond PD-L1 inhibition does not necessarily lead to better outcomes in ES-SCLC. The highly immunosuppressive tumor microenvironment and low baseline T-cell infiltration characteristic of SCLC may limit the efficacy of multi-checkpoint strategies. Survival estimation using RMST analysis strengthens our findings. Unlike the hazard ratio, the RMST provides an absolute and clinically interpretable measure of survival benefit over a defined time horizon and does not rely on the proportional hazard assumption. In addition, the RMST accounts for the entire curve within that timeframe, providing a more accurate representation of long-term plateaus. The approximately four-month improvement in the RMST observed with serplulimab compared to chemotherapy, as well as the improvement of 1.8–2.3 months over atezolizumab and durvalumab, may represent a clinically significant difference in a disease that has traditionally had a poor prognosis. Our results are consistent with recent network meta-analyses that ranked serplulimab among the most effective first-line chemoimmunotherapy regimens for ES-SCLC; our findings further support its favorable survival profile while extending the existing evidence through reconstructed individual patient data and RMST-based analyses, which enable evaluation of the entire survival trajectory and provide treatment effect estimates that are less dependent on the proportional hazards assumption.

However, several limitations must be acknowledged. First, this is an indirect comparison based on reconstructed individual patient data rather than original datasets. Although the IPDfromKM methodology has been validated and shown to closely reproduce published Kaplan–Meier curves, digitization and reconstruction may still introduce minor inaccuracies [25,26]. Secondly, the possible presence of cross-trial heterogeneity in baseline characteristics, including ethnicity, smoking status, platinum agent selection, and prevalence of brain metastases, has been addressed by testing for heterogeneity in the OS of the common comparator. However, the similarity observed in the control-arm OS should be interpreted cautiously and cannot alone establish exchangeability among trial populations. Thirdly, unanchored comparisons, particularly those involving the tiragolumab-containing regimen, are inherently less robust and should be interpreted with caution.

Toxicity outcomes were not formally analyzed in the present study because available evidence suggests that most adverse events in these regimens are primarily related to the chemotherapy backbone rather than to the immune checkpoint inhibitor itself. Across the included trials, the safety profiles appeared broadly comparable in terms of both frequency and type of adverse events, including immune-related toxicities among agents targeting the PD-1/PD-L1 pathway. Furthermore, immune-related adverse events are currently well recognized and routinely managed in clinical practice. In the setting of ES-SCLC, where the major unmet need remains the achievement of meaningful clinical benefit, treatment selection is therefore more commonly driven by efficacy considerations than by differences in toxicity profiles.

From a clinical perspective, our findings suggest that not all PD-1/PD-L1 inhibitors provide the same benefits for patients with ES-SCLC. If these differences are confirmed in prospective head-to-head trials or robust network meta-analyses, they could have implications for treatment selection, reimbursement decisions, and guideline development. However, given the methodological constraints of indirect comparisons, our results should be interpreted as hypothesis-generating rather than practice-changing. In parallel, analyses of real-world evidence incorporating large, multi-regional cohorts may help to validate the observed signals of relative effectiveness and could also provide comparative toxicity data, which was excluded from our analysis but is clinically significant.

## 5. Conclusions

In conclusion, this IPD-based indirect comparison suggests that, among currently available first-line ICI-based regimens for ES-SCLC, serplulimab plus chemotherapy may provide the greatest OS benefit, while durvalumab and atezolizumab remain effective standards of care. These findings contribute to the evolving therapeutic landscape of ES-SCLC, highlighting the need for direct comparative studies to definitively determine the most effective immunotherapy strategy for this aggressive disease.

## Figures and Tables

**Table 1:** Baseline characteristics of patients enrolled in the included randomized controlled trials. For each study, the active and control treatment arms are described, along with the sample size, proportion of smokers, proportion of Asian patients, and prevalence of baseline brain metastases. n: number of patients; %: percentage.

Trial [Reference]	Treatment Arms	n. of Pts	% Asian Pts	% Brain Metastases	% Smokers
ASTRUM-005 [20]	serplulimab + chemotherapy	389	67%	13%	26%
	vs. placebo + chemotherapy	196	71%	14%	24.5%
CASPIAN [17]	durvalumab + chemotherapy	268	13%	10%	45%
	vs. placebo + chemotherapy	269	16%	10%	47%
CA184-156 [14]	ipilimumab + chemotherapy	478	23%	12%	92%
	vs. placebo + chemotherapy	476	22%	10%	92%
KEYNOTE604 [13]	pembrolizumab + chemotherapy	228	23%	14%	65%
	vs. placebo + chemotherapy	225	14%	10%	59%
IMpower133 [11]	atezolizumab + chemotherapy	201	-	8.5%	37%
	vs. placebo + chemotherapy	202	-	9%	37%
SKYSCRAPER-02 [18]	tiragolumab + atezolizumab + chemotherapy	243	26%	19%	33%
	vs. atezolizumab + chemotherapy	247	27%	18.6%	31%

**Table 2:** Inter-treatment comparison between the first-line regimen for ES-SCLC. Statistically significant hazard ratio (HR) values with 95% confidence intervals (CIs) are reported.

Treatment Comparison	HR	Lower 95% CI	Upper 95% CI
serplulimab vs. durvalumab	0.736	0.596	0.910
serplulimab vs. ipilimumab	0.566	0.469	0.684
serplulimab vs. pembrolizumab	0.658	0.527	0.821
serplulimab vs. atezolizumab	0.780	0.642	0.949
serplulimab vs. tiragolumab + atezolizumab	0.709	0.570	0.881
durvalumab vs. ipilimumab	0.769	0.633	0.934
atezolizumab vs. ipilimumab	0.726	0.609	0.866

**Table 3:** Restricted mean survival time (RMST) analysis of OS for first-line regimen in patients with ES-SCLC. RMST values in months are reported together with 95% confidence intervals (CIs). RMST difference between active arms and chemotherapy controls or serplulimab + chemotherapy is reported and expressed in months.

Treatment Arm	RMST	Lower 95% CI	Upper 95% CI	Months Gained Compared to Chemotherapy Controls	Months Gained Compared to Serplulimab + Chemotherapy
controls	12.59	12.18	13.00	-	−4.1
serplulimab + chemotherapy	16.73	15.82	17.64	4.1	0
durvalumab + chemotherapy	14.44	13.40	15.49	1.8	−2.3
ipilimumab + chemotherapy	12.87	12.21	13.53	0.3	−3.8
pembrolizumab + chemotherapy	13.59	12.40	14.79	1	−3.1
atezolizumab + chemotherapy	14.91	14.08	15.74	2.3	−1.8
tiragolumab + atezolizumab + chemotherapy	14.30	13.22	15.37	1.7	−2.4

## Data Availability

The data presented in this study are available in the Supplementary Materials which include an Excel file (“sclcfig2.xls”) with individual patient data and a PDF file (“Supplementary_material.pdf”) explaining the codes employed in the Excel file.

## Supplementary Materials

The following supporting information can be downloaded at: https://www.mdpi.com/article/10.3390/cancers18121869/s1, Supplementary Materials S1. PRISMA_2020_checklist; Supplementary Materials S2. codes; Supplementary Materials S3. sclcfig2.

## Abbreviations

The following abbreviations are used in this manuscript:

CI	Confidence Interval
ES-SCLC	Extensive-Stage Small-Cell Lung Cancer
HR	Hazard Ratio
ICI	Immune Checkpoint Inhibitor
IPD	Individual Patient Data
KM	Kaplan–Meier
OS	Overall Survival
RCT	Randomized Controlled Trial
RMST	Restricted Mean Survival Time

## Appendix A

**Table A1:** Comparison of hazard ratios derived from reconstructed individual patient data and those reported in the original randomised controlled trials. Hazard ratios (HRs) with 95% confidence intervals (CIs) were obtained using two approaches: (1) reconstruction of individual patient data (IPD) from published Kaplan–Meier curves using the IPDfromKM algorithm, and (2) direct extraction from the original published randomised controlled trial (RCT) reports. Abbreviations: CI, confidence interval; HR, hazard ratio; IPD, individual patient data; RCT, randomised controlled trial.

Trial	HR with 95% CI Reported in the Original RCT	HR with 95% CI Determined from Reconstructed IPD
ASTRUM-005	HR = 0.62 (95% CI 0.50–0.76)	HR = 0.63 (95% CI 0.51–0.77)
CASPIAN	HR = 0.75 (95% CI 0.62–0.91)	HR = 0.75 (95% CI 0.62–0.91)
CA184-156	HR = 0.94 (95% CI 0.81–1.09)	HR = 0.92 (95% CI 0.79–1.06)
KEYNOTE-604	HR = 0.80 (95% CI 0.64–0.98)	HR = 0.80 (95% CI 0.65–0.99)
IMpower133	HR = 0.76 (95% CI 0.60–0.95)	HR = 0.76 (95% CI 0.60–0.95)
SKYSCRAPER-02	HR = 1.09 (95% CI 0.88–1.35)	HR = 1.11 (95% CI 0.90–1.38)

## References

[1] Oronsky B., Reid T.R., Oronsky A., Carter C.A. (2017). What’s New in SCLC? A Review. Neoplasia.

[2] Wang S., Tang J., Sun T., Zheng X., Li J., Sun H., Zhou X., Zhou C., Zhang H., Cheng Z. (2017). Survival changes in patients with small cell lung cancer and disparities between different sexes, socioeconomic statuses and ages. Sci. Rep..

[3] Micke P., Faldum A., Metz T., Beeh K.M., Bittinger F., Hengstler J.G., Buhl R. (2002). Staging small cell lung cancer: Veterans Administration Lung Study Group versus International Association for the Study of Lung Cancer—What limits limited disease?. Lung Cancer.

[4] Ganti A.K.P., Loo B.W., Bassetti M., Blakely C., Chiang A., D’Amico T.A., D’Avella C., Dowlati A., Downey R.J., Edelman M. (2021). Small Cell Lung Cancer, Version 2.2022, NCCN Clinical Practice Guidelines in Oncology. J. Natl. Compr. Cancer Netw..

[5] Byers L.A., Rudin C.M. (2015). Small cell lung cancer: Where do we go from here?. Cancer.

[6] Meijer J.J., Leonetti A., Airò G., Tiseo M., Rolfo C., Giovannetti E., Vahabi M. (2022). Small cell lung cancer: Novel treatments beyond immunotherapy. Semin. Cancer Biol..

[7] Dingemans A.C., Früh M., Ardizzoni A., Besse B., Faivre-Finn C., Hendriks L.E., Lantuejoul S., Peters S., Reguart N., Rudin C.M. (2021). Small-cell lung cancer: ESMO Clinical Practice Guidelines for diagnosis, treatment and follow-up. Ann. Oncol..

[8] Wang S., Zimmermann S., Parikh K., Mansfield A.S., Adjei A.A. (2019). Current Diagnosis and Management of Small-Cell Lung Cancer. Mayo Clin. Proc..

[9] Zugazagoitia J., Paz-Ares L. (2022). Extensive-Stage Small-Cell Lung Cancer: First-Line and Second-Line Treatment Options. J. Clin. Oncol..

[10] Saito M., Shiraishi K., Goto A., Suzuki H., Kohno T., Kono K. (2018). Development of targeted therapy and immunotherapy for treatment of small cell lung cancer. Jpn. J. Clin. Oncol..

[11] Horn L., Mansfield A.S., Szczęsna A., Havel L., Krzakowski M., Hochmair M.J., Huemer F., Losonczy G., Johnson M.L., Nishio M. (2018). First-Line Atezolizumab plus Chemotherapy in Extensive-Stage Small-Cell Lung Cancer. N. Engl. J. Med..

[12] Cheng Y., Han L., Wu L., Chen J., Sun H., Wen G., Ji Y., Dvorkin M., Shi J., Pan Z. (2022). Effect of First-Line Serplulimab vs. Placebo Added to Chemotherapy on Survival in Patients with Extensive-Stage Small Cell Lung Cancer: The ASTRUM-005 Randomized Clinical Trial. JAMA.

[13] Rudin C.M., Awad M.M., Navarro A., Gottfried M., Peters S., Csőszi T., Cheema P.K., Rodriguez-Abreu D., Wollner M., Yang J.C. (2020). Pembrolizumab or Placebo Plus Etoposide and Platinum as First-Line Therapy for Extensive-Stage Small-Cell Lung Cancer: Randomized, Double-Blind, Phase III KEYNOTE-604 Study. J. Clin. Oncol..

[14] Reck M., Luft A., Szczesna A., Havel L., Kim S.W., Akerley W., Pietanza M.C., Wu Y.L., Zielinski C., Thomas M. (2016). Phase III Randomized Trial of Ipilimumab Plus Etoposide and Platinum Versus Placebo Plus Etoposide and Platinum in Extensive-Stage Small-Cell Lung Cancer. J. Clin. Oncol..

[15] Paz-Ares L., Dvorkin M., Chen Y., Reinmuth N., Hotta K., Trukhin D., Statsenko G., Hochmair M.J., Özgüroğlu M., Ji J.H. (2019). Durvalumab plus platinum-etoposide versus platinum-etoposide in first-line treatment of extensive-stage small-cell lung cancer (CASPIAN): A randomised, controlled, open-label, phase 3 trial. Lancet.

[16] Liu S.V., Reck M., Mansfield A.S., Mok T., Scherpereel A., Reinmuth N., Garassino M.C., De Castro Carpeno J., Califano R., Nishio M. (2021). Updated Overall Survival and PD-L1 Subgroup Analysis of Patients with Extensive-Stage Small-Cell Lung Cancer Treated with Atezolizumab, Carboplatin, and Etoposide (IMpower133). J. Clin. Oncol..

[17] Goldman J.W., Dvorkin M., Chen Y., Reinmuth N., Hotta K., Trukhin D., Statsenko G., Hochmair M.J., Özgüroğlu M., Ji J.H. (2021). Durvalumab, with or without tremelimumab, plus platinum-etoposide versus platinum-etoposide alone in first-line treatment of extensive-stage small-cell lung cancer (CASPIAN): Updated results from a randomised, controlled, open-label, phase 3 trial. Lancet Oncol..

[18] Rudin C.M., Liu S.V., Soo R.A., Lu S., Hong M.H., Lee J.S., Bryl M., Dumoulin D.W., Rittmeyer A., Chiu C.H. (2024). SKYSCRAPER-02: Tiragolumab in Combination with Atezolizumab Plus Chemotherapy in Untreated Extensive-Stage Small-Cell Lung Cancer. J. Clin. Oncol..

[19] Zhang Y., Wei R., Song G., Yang X., Zhang M., Liu W., Xiong A., Zhang X., Li Q., Yang W.J. (2024). Insights into the mechanisms of serplulimab: A distinctive anti-PD-1 monoclonal antibody, in combination with a TIGIT or LAG3 inhibitor in preclinical tumor immunotherapy studies. mAbs.

[20] Cheng Y., Zhang S., Han L., Wu L., Chen J., Zhao P., Sun H., Wen G., Ji Y., Zimina A. (2025). First-line serplulimab plus chemotherapy in extensive-stage small-cell lung cancer: Updated results and biomarker analysis from the ASTRUM-005 randomized clinical trial. Cancer Commun..

[21] Liu N., Zhou Y., Lee J.J. (2021). IPDfromKM: Reconstruct individual patient data from published Kaplan-Meier survival curves. BMC Med. Res. Methodol..

[22] Messori A. (2021). Synthetizing Published Evidence on Survival by Reconstruction of Patient-Level Data and Generation of a Multi-Trial Kaplan-Meier Curve. Cureus.

[23] Damuzzo V., Rivano M., Baldo P., Cancanelli L., Di Spazio L., Ossato A., Chiumente M., Messori A., Mengato D. (2022). Applicazione dell’intelligenza artificiale per un precoce confronto di efficacia tra nuovi farmaci oncologici. Recent. Progress. Med..

[24] Page M.J., McKenzie J.E., Bossuyt P.M., Boutron I., Hoffmann T.C., Mulrow C.D., Shamseer L., Tetzlaff J.M., Akl E.A., Brennan S.E. (2021). The PRISMA 2020 statement: An updated guideline for reporting systematic reviews. BMJ.

[25] Najah Q., Makhlouf H.A., Osman A.S.A., Almosilhy N.A., Abusalah M.A., Abuhlaiga M.A., Qutob I.A., Abdelgawad H.A.H. (2026). Efficacy and tolerability of first-line treatment regimens for extensive-stage small cell lung cancer—A grade-assessed network meta-analysis of randomized controlled trials. BMC Cancer.

[26] Rogula B., Lozano-Ortega G., Johnston K.M. (2022). A Method for Reconstructing Individual Patient Data From Kaplan-Meier Survival Curves That Incorporate Marked Censoring Times. MDM Policy Pract..

