# Supplementary material for: Comparative Efficacy of First-Line Therapeutic Options for ES-SCLC: An Indirect Comparison Using IPDfromKM-Reconstructed Individual Patient Data
Source: Cancers (Basel). 2026 Jun 8;18(12):1869. doi: 10.3390/cancers18121869 (PMC13297242; doi:10.3390/cancers18121869)
Supplement: Supplementary file 1 [file cancers-18-01869-s001.zip › supplementary_material-updated/Supplementary Materials 2. codes.pdf]

SUPPLEMENTARY MATERIAL

The enclosed Excel file (named “sclcfig2.xls”) contains the reconstructed patient-level dataset of the Kaplan-Meier curves shown in Figure 2 of the article (see below).

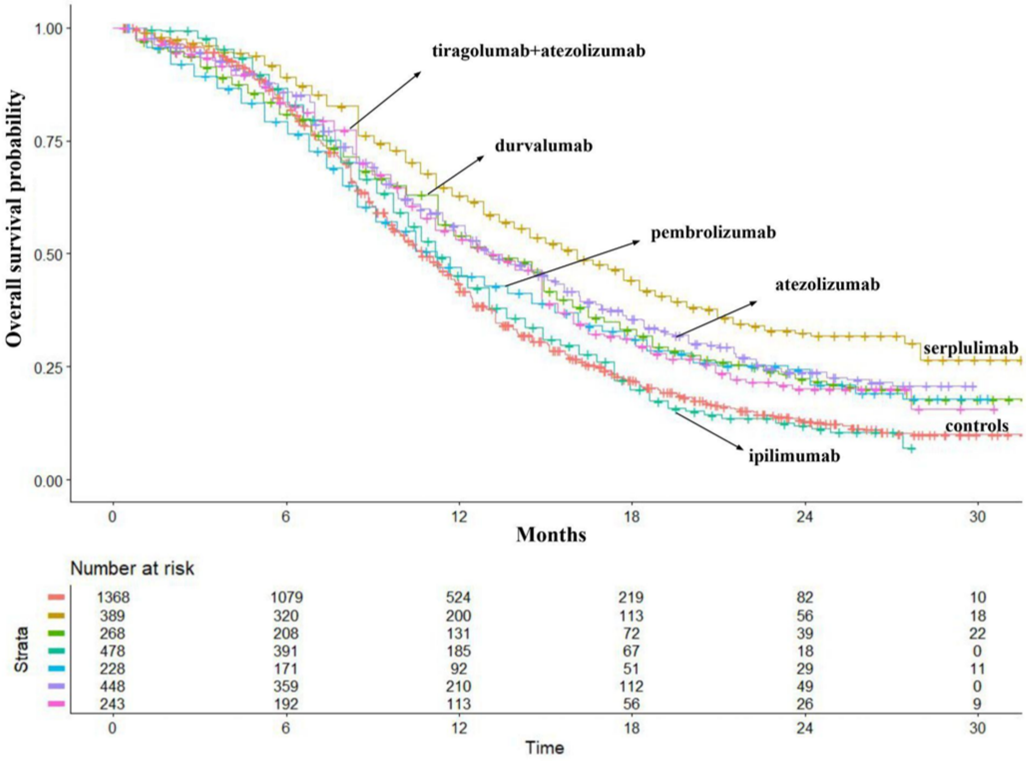

Figure 2. OS comparison of first-line treatments for ES-SCLC. After reconstructing individual patient data (IPD) from six trials, the following OS KM curves were generated for active arms of the RCTs, which combine platinum-based chemotherapy with serplulimab (n = 389; in gold); durvalumab (n = 268; in light green); ipilimumab (n = 478; in dark green); pembrolizumab (n = 228; in light blue); atezolizumab (n = 448, in violet); and tiragolumab + atezolizumab (n = 243; in pink). The control arm (n = 1358; in red) was generated by pooling IPD from the chemotherapy-based control arms. Abbreviations: n, number of patients.

**Numerical code in the Excel file (column RX)**	**Legend**
1	control arm generated by pooling IPD from the chemotherapy-based control arms (n = 1368; in red).
2	platinum-based chemotherapy with serplulimab (n = 389; in gold);
3	durvalumab (n = 268; in light green);
4	ipilimumab (n = 478; in dark green);
5	pembrolizumab (n = 228; in light blue);
6	atezolizumab (n = 448, in violet);
7	tiragolumab + atezolizumab (n = 243; in pink).
Note: in the column named STATUS of the Excel file, outcomes are coded 1 for patients with event and 0 for patients without event while the column named SURVIVAL_T reports the length of the follow-up (in months) for individual patients.	
